# Dissection of *Paenibacillus polymyxa* NSY50-Induced Defense in Cucumber Roots against *Fusarium oxysporum* f. sp. *cucumerinum* by Target Metabolite Profiling

**DOI:** 10.3390/biology11071028

**Published:** 2022-07-08

**Authors:** Nanshan Du, Qian Yang, Hui Guo, Lu Xue, Ruike Fu, Xiaoxing Dong, Han Dong, Zhixin Guo, Tao Zhang, Fengzhi Piao, Shunshan Shen

**Affiliations:** 1 College of Horticulture, Henan Agricultural University, Zhengzhou 450002, China; fangshan711@163.com (N.D.); yangyangqian023@163.com (Q.Y.); gh10242021@163.com (H.G.); 18860352800@163.com (L.X.); 18838978252@163.com (R.F.); wuxian_mige@163.com (X.D.); 440069@henau.edu.cn (H.D.); guozhixin666@163.com (Z.G.); zhangtao3375@163.com (T.Z.); piao1203@163.com (F.P.); 2 College of Plant Protection, Henan Agricultural University, Zhengzhou 450002, China

**Keywords:** Fusarium wilt, *P. polymyxa* NSY50, metabolomics, GSH cycle, cucumber

## Abstract

**Simple Summary:**

Plant growth-promoting rhizobacteria (PGPR) have significant potential to enhance the tolerance of biotic and abiotic stresses and the productivity of crops. However, the mechanism of PGPR in improving plant resistance to pathogens is unclear. Recently, the newly isolated *Paenibacillus polymyxa* strain NSY50 was shown to considerably suppress the Fusarium wilt of cucumber plants. This study was carried out to explore the underlying mechanism of NSY50 in improving plant resistance to pathogen invasion via target metabolite profiling, and the results indicated that strain NSY50 was able to alleviate Fusarium wilt stress by activating GSH metabolism and improving redox balance. Our research findings enable a deeper understanding of *P. polymyxa* NSY50-induced enhanced defense against *F. oxysporum* in cucumber.

**Abstract:**

To gain insights into the roles of beneficial PGPR in controlling soil-borne disease, we adopted a metabolomics approach to investigate the beneficial impacts of *P. polymyxa* NSY50 on cucumber seedling roots under the pathogen of *Fusarium oxysporum* f. sp. *cucumerinum* (FOC). We found that NSY50 pretreatment (NSY50 + FOC) obviously reduced the production of reactive oxygen species (ROS). Untargeted metabolomic analysis revealed that 106 metabolites responded to NSY50 and/or FOC inoculation. Under FOC stress, the contents of root osmotic adjustment substances, such as proline and betaine were significantly increased, and dehydroascorbic acid and oxidized glutathione (GSH) considerably accumulated. Furthermore, the contents of free amino acids such as tryptophan, phenylalanine, and glutamic acid were also significantly accumulated under FOC stress. Similarly, FOC stress adversely affected glycolysis and the tricarboxylic acid cycles and transferred to the pentose phosphate pathway. Conversely, NSY50 + FOC better promoted the accumulation of α-ketoglutaric acid, ribulose-5-phosphate, and 7-phosphosodiheptanone compared to FOC alone. Furthermore, NSY50 + FOC activated GSH metabolism and increased GSH synthesis and metabolism-related enzyme activity and their encoding gene expressions, which may have improved redox homoeostasis, energy flow, and defense ability. Our results provide a novel perspective to understanding the function of *P. polymyxa* NSY50, accelerating the application of this beneficial PGPR in sustainable agricultural practices.

## 1. Introduction

As one of the most important vegetable crops worldwide, cucumber (*Cucumis sativus* L.) is seriously threatened by Fusarium wilt caused by the soil-borne fungal pathogen *Fusarium oxysporum* f. sp. *cucumerinum* (FOC) [1], a ruinous vascular disease that typically leads to reduced cucumber yield and has incurred huge economic losses [2,3]. The pathogen is difficult to control due to its broad host range and its ability to survive in soil and seeds for a number of years or decades [4]. Given the lack of available effective chemical products and resistant cultivars, the non-target impacts of pesticides are harmful to the environment and pose potential health risks [5]. These challenges have prompted research into ecofriendly cropping strategies, with a strong emphasis on utilizing cost-effective and environmentally friendly farming methods to improve crop endurance and stress tolerance [6,7,8].

To date, the biological control of plant diseases utilizing the disease-suppressive effects of plant growth-promoting rhizobacteria (PGPR) has been efficiently documented and suggested to control diseases and improve growth parameters in a diverse of vegetable crops [9,10,11,12,13]. *Paenibacillus polymyxa* is a soil-dwelling, nonpathogenic, endospore-forming bacterium frequently associated with the roots of higher plants [14]. This Bacillus species is important due to its ability to activate plant growth-promoting hormones, enhance phosphorus and iron absorption in plants, and induce systemic resistance in plants [15,16,17]. Furthermore, *P. polymyxa* strains are capable of producing diverse hydrolytic enzymes and antibiotics, such as cellulases, xylanase, proteases, β-1,3-glucanases, chitinases, polymyxins, and fusaricidins, which act in essential roles in the eradication of plant pathogens [18,19,20]. Due to their wide range of plant hosts, these strains have significant potential as biological-control agents against a diverse array of pathogens such as *Botrytis cinerea*, *Fusarium oxysporum*, *Phytophthora palmivora*, *Pseudomonas syringae* and *Ralstonia solanacearum* [14,21,22,23,24].

Multiple genomics techniques have recently been applied to comprehensively elucidate the mechanisms of plant–PGPR interactions [9,25,26,27,28]. As the end-products of gene expression, metabolites delegate all levels of regulation between genes and enzymes, and they can be applied to gain a comprehensive dataset on a plant’s response to both PGPR and stress [9]. So far, little attention has been paid to the metabolomic changes in cucumbers inoculated with *P. polymyxa* under an FOC attack.

In our previous studies, we demonstrated the ability of *P. polymyxa* NSY50 to reduce disease severity. Various effective mechanisms, such as the induction of systemic resistance [21], the regulation of the rhizospheric microbial community [3], and the activation of defense-associated proteins [26], may contribute an important role in the efficacy of biocontrol against Fusarium wilt of cucumber. Therefore, the objectives of this research were to gain insight into the metabolomic response of cucumber plants inoculated with *P. polymyxa* NSY50 under Fusarium wilt stress and to highlight the inherent defense mechanism of cucumber plants by stimulating metabolic pathways. Here, we identified a total of 108 metabolites that are down- or up-regulated by the treatment of *P. polymyxa* NSY50 and/or FOC through the utilization of liquid chromatography (LC)–MS and gas chromatography–mass spectrometry (GC–MS) analyses. To strengthen the metabolic findings, we determined physiological parameters, such as biomass, soluble protein content, and lipid peroxidation. We also tested the different types of enzymes activities along with their encoding gene expressions in cucumber plants. Our results provide new insights into the metabolomic mechanisms of *P. polymyxa* NSY50 function, accelerating the application of this beneficial PGPR in sustainable agricultural practices.

## 2. Materials and Methods

### 2.1. Microbial Culture Conditions, Plant Material and Treatments

*P.polymyxa*-NSY50 was cultured in LB broth for 72 h at 28 °C. Cell density in suspensions were set to 10^8^ cells per mL. The cucumber Fusarium wilt pathogen FOC was isolated and prepared according to our previous work [3,21].

Uniform cucumber seeds (*Cucumis sativus* L. cv. “Jinchun NO.4”) were surface-sterilized with 5% ethanol and placed in the dark for germination. The germinated seeds were sown in a 50-hole tray filled with vermiculite growth substrate. The seedlings were transplanted into 4 L plastic containers after their cotyledons had fully expanded, at which point we added a half-strength Hoagland solution. In each container, three seedlings were placed. The seedlings were cultivated in an artificial-light growth chamber at Henan Agricultural University, and the growth environments were maintained as follows: 28/25 °C (day/night) temperature, relative humidity of 65–75%, light intensity of 200 µmol m^−2^ s^−1^, and a 14/10 h day/night photoperiod. Cucumber seedlings at the two-leaf stage were used for the following treatments. (1) Control: cucumber seedlings grown in Hoagland solution; (2) NSY50: cucumber seedlings grown in 100 mL of a 1.0 × 10^8^ CFU/mL NSY50 cell suspension containing a half-strength Hoagland nutrient solution; (3) FOC: a pre-cultured (6 days) 100 mL cell suspension of FOC (1 × 10^7^ conidia/mL) was added into the tanks; and (4) NSY50+ FOC: a pre-cultured (3 days) 100 mL NSY50 (1 × 10^8^ CFU/mL) solution was inoculated and 3 days later again inoculated with a 100 mL cell suspension of FOC (1 × 10^7^ conidia/mL). Each treatment contained four container seedlings, which were assembled in a completely randomized design with repetition in triplicates, yielding a total of 36 seedlings per treatment. The nutrient solution was constantly aerated with an air pump and replaced every three days. Cucumber roots were harvested at 1-, 3- and 5-days post-inoculation (dpi) with the pathogen. Following 7 days of FOC treatment, the fresh weight of plant was measured with three biological replicates after washing and drying with gauze. To calculate the dry weights, the plant samples were first dried at 105 °C for 15 min and then kept at 75 °C until their constant weights.

### 2.2. Estimation of MDA Content and O_2_^.-^ Production Rate

Roots were collected at 1-, 3- and 5-days following treatment with FOC. The content of MDA in the roots of cucumber was determined as thiobarbituric acid reactive substance formation, as described by Liu et al. [29]. The rate of superoxide anion formation was calculated using the hydroxylamine reaction method developed by Elstner and Heupel [30]. The generation rate of O_2_^.-^ is expressed in nmol min^−1^ g^−1^ FW.

### 2.3. Determination of H_2_O_2_ and Soluble Protein Contents and Histochemical Detection of H_2_O_2_ and O_2_^.-^ in Roots

The content of H_2_O_2_ in roots of cucumber was determined as described by Yuan et al. [31] with some modifications. Briefly, 0.5 g root tissues were macerated in 2 mL of ice-cold acetone before being centrifuged at 10,000× *g* at 4 °C for 10 min. Around 1 mL of supernatant was then mixed with 0.2 mL of concentrated ammonia and 0.1 mL of 20% TiCl_4_ followed by centrifugation at 8000× *g* for 15 min. Finally, 2 mL of 2 M H_2_SO_4_ was added in the deposition, and absorbance was read at 415 nm. The content of H_2_O_2_ in root is expressed in µmol·g^−1^ FW.

For the measurement of the soluble protein content of the roots, fresh roots (0.5 g) were ground with 5 mL of a 0.05 M phosphate buffer (pH 6.7) before being centrifuged at 10,000× *g* for 10 min at 4 °C. Then and the collected supernatant was then used for protein analysis according to the method of Bradford [32], and the protein content was measured using a standard curve made from bovine serum albumin.

The histochemical staining analysis of cucumber roots was carried out 3 days after the infection with FOC. For O_2_^.-^ visualization, roots were cut into small pieces including the root tip (1 cm) and infiltrated with a 10 mM K-citrate buffer (pH 6.0) solution containing 0.5 mM nitroblue tetrazolium (NBT) following the method described by Frahry and Schopfer [33]. Likewise, H_2_O_2_ accumulation was visualized via 3,3-diaminobenzidine (DAB) staining, as previously described by Guo et al. [34], and the stained roots were photographed with a Leica DM2500 camera (Leica Microsystems, Wetzlar, HE, Germany).

### 2.4. Metabolite Analyses

Both GC–MS and ultrahigh-performance LC (UPLC)–MS (LC–MS) techniques were used for untargeted metabolomic analysis. The roots of cucumber seedlings were prepared for metabolism analysis at 3 dpi of FOC. For GC–MS metabolite analyses, six independent biological replications were randomly selected. Cucumber root samples (60 mg each) were separately extracted with 40 µL of L-2-chlorophenylalanine (0.3 mg/mL stock in methanol) and 360 µL of cold methanol, as described by Liu et al. [35].

For LC–MS metabolism analysis, three independent biological replications of each treatment were analyzed. We transferred 30 mg samples to 1.5 mL centrifuge tubes, to which 580 µL of a mixed solution (methanol/water= 6:4 (*v*/*v*)) and 20 µL 2-chloro-l-phenylalanine (0.3 mg/mL, dissolved in methanol) were then added, and the samples were homogenized with a TissueLyser (TissueLyser-192, Shanghai Jingxin Industrial Development CO., LTD, Shanghai, China). Next, the tissue homogenate was obtained by sonication in an ice-water bath for 10 min, followed by the addition of 150 µL of chloroform and vortexing for 1 min. Next, the samples were stored in a refrigerator at −20 °C for 30 min followed by centrifugation at 13,000× *g* rpm for 10 min (4 °C). We transferred 500 µL aliquots to new tubes, then dried the aliquots using a freeze concentration centrifugal dryer. The dried pellets were re-dissolved with 250 µL of a mixed solution (water/methanol = 1:1 (*v*/*v*)). Following centrifugation for 15 min (4 °C) at 13,000 *g* rpm, we transferred (180 µL) supernatants to LC vials and kept them at −20 °C until LC–MS analysis.

LC–MS analysis was carried out on a Waters UPLC I-class system mechanized with a binary solvent delivery manager and a sample manager connected to a Waters VION IMS Q-TOF Mass Spectrometer with an electrospray interface (Waters Corporation, Milford, CT, USA). Each 3 µL supernatant of the derivatized solution was injected, followed by separation on an Acquity UPLC BEH C18 column (100 mm × 2.1 mm i.d., 1.7 µm; Waters, Milford, CT, USA). The temperature of the column was maintained at 45 °C, and separation was accomplished through the utilization of the following gradients: 5–25 % B over 0–1.5 min, 25–90 % B over 1.5–10.0 min, and 90% B over 10–13 min. The concentration was retained at 5% B for 2 min at a flow rate of 0.40 mL/min, where B is 10 mM ammonium acetate (pH = 9), and A is acetonitrile–10 mM ammonium acetate (pH = 9) (*v*/*v* = 9:1). The mass spectrometric data were extracted with a Waters VION IMS Q-TOF Mass Spectrometer equipped with an electrospray ionization source working in either the positive or negative ion mode. The source and desolvation temperatures were set at 120 °C and 500 °C, respectively, and the flow rate of desolvation gas was 900 L/h. Centroid data were collected from 50 *m/z* to 1000 *m/z* with a scan time of 0.1 s and an interscan delay of 0.02 s over a 13 min analysis time.

The acquired MS data from GC–MS and LC–MS were analyzed with the metabolomics processing software Progenesis QI v2.3 software (Waters Corporation, Milford, CT, USA) for baseline filtering, peak identification, integration, and retention, as described by Zhang et al. [36]. Briefly, the main parameters were as follows: precursor tolerance of 5 ppm, product tolerance of 10 ppm, product ion threshold of 5%, and retention time tolerance of 0.02 min. The processed data were identified and annotated with libraries of QI and public databases including the Human Metabolome Database (http://www.hmdb.ca/ (accessed on 5 February 2020)) and LIPID MAPS (http://www.lipidmaps.org/ (accessed on 5 February 2020)). Internal standard peaks, as well as any known false-positive peaks (including noise, column bleed, and derivatized reagent peaks), were removed from the data matrix, and the peaks from the same metabolite were combined. The criterion for the screening of differential metabolites was arbitrarily set as the following standard: *p* < 0.05 and fold change > 1.5.

### 2.5. Measurements of GSH Content and Enzyme Activities

The GSH content was determined in accordance with the work of Zhong et al. [37]. Approximately 300 mg of composite cucumber root tissue was ground in 2 mL of 6% metaphosphoric acid containing 2 mM Ethylene Diamine Tetraacetic Acid (EDTA) followed by centrifugation at 12,000× *g* and 4 °C for 10 min. Then, the supernatant was collected for the measurement of the total GSH and oxidized GSH (GSSG) by the 5,5′-dithio-bis (2-nitrobenzoicacid)-GSSG reductase recycling method. The GSH content was then calculated by subtracting total GSH from GSSG.

For the measurement of GSH reductase (GR) activity, composite root samples (0.3 g) were ground in 3 mL of an ice-cold 25 mM HEPES buffer (pH 7.8). The homogenates were then centrifuged at 12,000× *g* for 20 min (4 °C), and the collected supernatants were used to evaluate the enzymatic activity. GR activity was estimated in accordance with the method of Halliwell and Foyer [38] method, and the rate of decrease NADPH absorbance at 340 nm was used to finally estimate the GR activity. The activity of GSH peroxidase (GPX) was measured following the method established by Quessada and Macheix [39]. Thioredoxin reductase (TrxR) activity was measured as described by Dhindsa et al. [40]. The γ-glutamylcysteine ligase (GCL) activity was determined from the rate of formation of inorganic phosphate (assumed to be equal to the rate of formation of ADP) using a GCL assay kit (Comin Biotechnology Co., Ltd. Suzhou, China) according to the manufacturer’s instructions, and the absorbance was measured at 660 nm.

### 2.6. RNA Extraction, and Gene Expression Analysis by qRT-PCR

A Quick RNA isolation kit (Hua Yue Yang, Beijing, China) was used to extract the total RNA from ground cucumber roots following the company’s instructions. Then, 1 µg of total RNA was reverse-transcribed into the cDNA template with the HiScript™ Q RT SuperMix (Vazyme, Nanjing, China) following the company’s instructions. A SYBR Green PCR Master Mix (Takara, Chiga, Japan) was used to perform reverse transcription (RT)-qPCR experiments. The RT-qPCR reaction time was maintained as: 3 min at 95 °C, followed by 40 cycles of 30 s at 95 °C, 30 s at 58 °C, and 1 min at 72 °C. The actin gene was employed as the internal control gene. The primer sequences were designed based on the corresponding gene sequence by searching the database of NCBI (https://www.ncbi.nlm.nih.gov/tools/primer-blast/ (accessed on 7 September 2020)), and Beacon Designer 7.9 (Premier Biosoft International, Palo Alto, CA, USA) was used to design the primers that are listed in Supplemental Table S1. Relative gene expressions were evaluated as described by Livak and Schmittgen [41].

### 2.7. Statistical Analysis

Prior to data analysis, the heat maps of all metabolites based on the relative peak area and other analytical data were normalized using SPSS 20.0 software. Hierarchical cluster analysis was performed with cluster software, and Java Treeview was used to visualize the resulting heat map. All experimental data were statistically analyzed with three biological replications, and results were statistically analyzed using SPSS 20.0 software (SPSS Inc., Chicago, IL, USA) and GraphPad Prism software version 5.0. The significant differences among the treatments were evaluated using Duncan’s multiple comparison test at the level of *p <* 0.05.

## 3. Results

### 3.1. Characteristics of Plant Growth under Different Treatments

**Figure 1:**
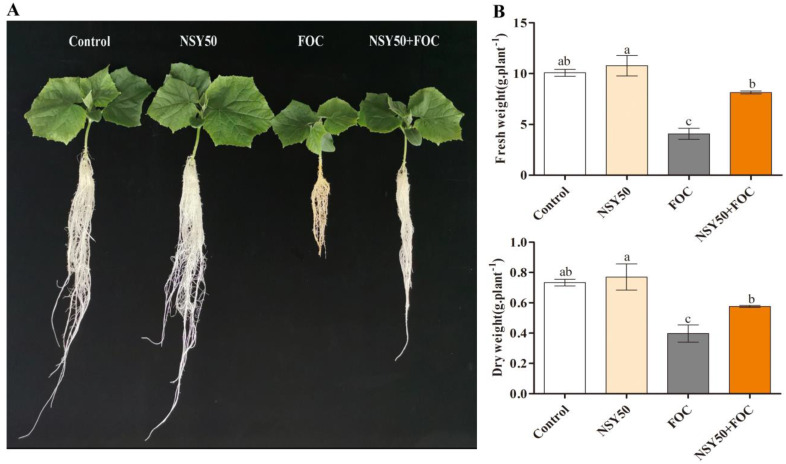
Effects of plant-growth-promoting bacteria-NSY50 on the growth of cucumber seedlings under Fusarium wilt stress. (**A**) Phenotypes images, (**B**) Fresh and dry weight. Values are the means ± SD, *n* = 3 (biological replicates), and different letters (a > b > c) indicate a significant difference at *p* < 0.05, as determined by Duncan’s multiple-range test.

The growth biomass of cucumber seedlings was evaluated after seven days post-inoculation (dpi) of the pathogen. As shown in Figure 1, without FOC inoculation, the addition of NSY50 promoted the growth of cucumber seedlings to a certain extent, and the fresh and dry weights were increased by 6.98% and 5.00%, respectively, compared to the untreated plant, but the difference was not statistically significant. However, inoculation with FOC markedly slowed the growth indices of cucumber seedlings and decreased fresh and dry weights by 59.64% and 45.91%, respectively, compared to the control. Cucumber seedlings pretreated with NSY50 and then inoculated with FOC (NSY50 + FOC) presented increased the growth attributes compared to those under the FOC treatment, revealing the significantly alleviated inhibitory effect of FOC on cucumber plant growth.

### 3.2. Lipid Peroxidation

**Figure 2:**
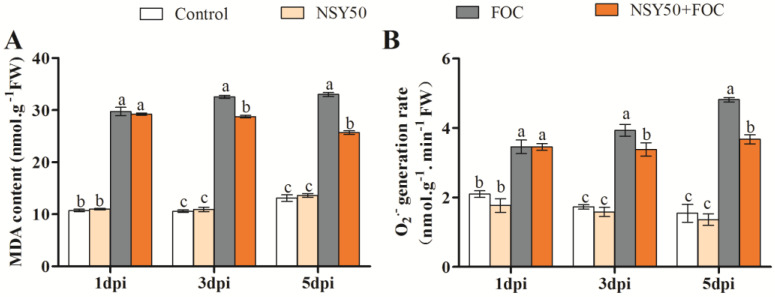
Effects of NSY50 on MDA content (**A**) and O_2_^.-^ generation rate (**B**) of cucumber seedling roots at 1-, 3- and 5-days post-inoculation (dpi) with FOC in different treatments. Each value is means ± SE of three independent experiments. Values are the means ± SD, *n* = 3 (biological replicates), and different letters (a > b > c) indicate a significant difference at *p* < 0.05, as determined by Duncan’s multiple-range test.

FOC stress alone significantly elevated the MDA content and O_2_^.-^ production rate compared to the control (Figure 2). At 1, 3 and 5 dpi, the root MDA contents of cucumber seedlings treated with FOC were 1.77, 2.07, and 1.52 times greater than the untreated plants, respectively, and the O_2_^.-^ production rates were 0.65, 1.27, and 2.11 times greater than the untreated and other treatments, respectively. Under NSY50 + FOC at 3 and 5 dpi, the MDA content of cucumber seedlings significantly decreased by 11.68% and 22.20%, respectively, compared to the FOC treatment. The O_2_^.-^ production rate also significantly decreased by 14.01% (3 dpi) and 23.65% (5 dpi). However, inoculation with NSY50 alone had no significant effects on the root MDA content and O_2_^.-^ production rate of cucumber seedlings.

**Figure 3:**
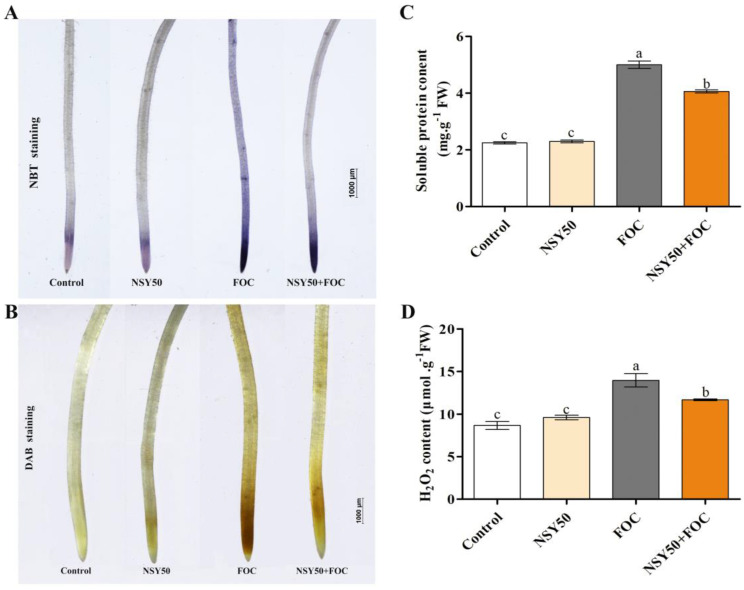
Effects of NSY50 on oxidative damage and soluble protein content in cucumber roots under Fusarium wilt stress at 3 days post-inoculation. (**A**) Histochemical staining by nitroblue tetrazolium (NBT) of O_2_^.-^, (**B**) Histochemical staining by 3, 3-Diaminobenzidine (DAB) of H_2_O_2_, (**C**) Soluble protein contents. (**D**) H_2_O_2_ contents. Values are the means ± SD, *n* = 3 (biological replicates), and different letters (a > b > c) indicate a significant difference at *p* < 0.05, as determined by Duncan’s multiple-range test.

To further elucidate the effect of NSY50 on the oxidative damage of cucumber roots under Fusarium wilt stress, we measured H_2_O_2_ and soluble protein contents in cucumber roots at 3 dpi with FOC, and the results were further verified by NBT and DAB. The results were consistent with the findings of the MDA content and rate of O_2_^.-^ production (Figure 3). The concentrations of soluble protein and H_2_O_2_ in the FOC treatment increased by 122.68% and 61.30%, respectively, compared to those of the control treatment. Furthermore, the soluble protein and H_2_O_2_ contents in the NSY50 + FOC treatment decreased by 18.77% and 16.46%, respectively, compared to the FOC treatment.

### 3.3. Metabolomic Analysis

**Figure 4:**
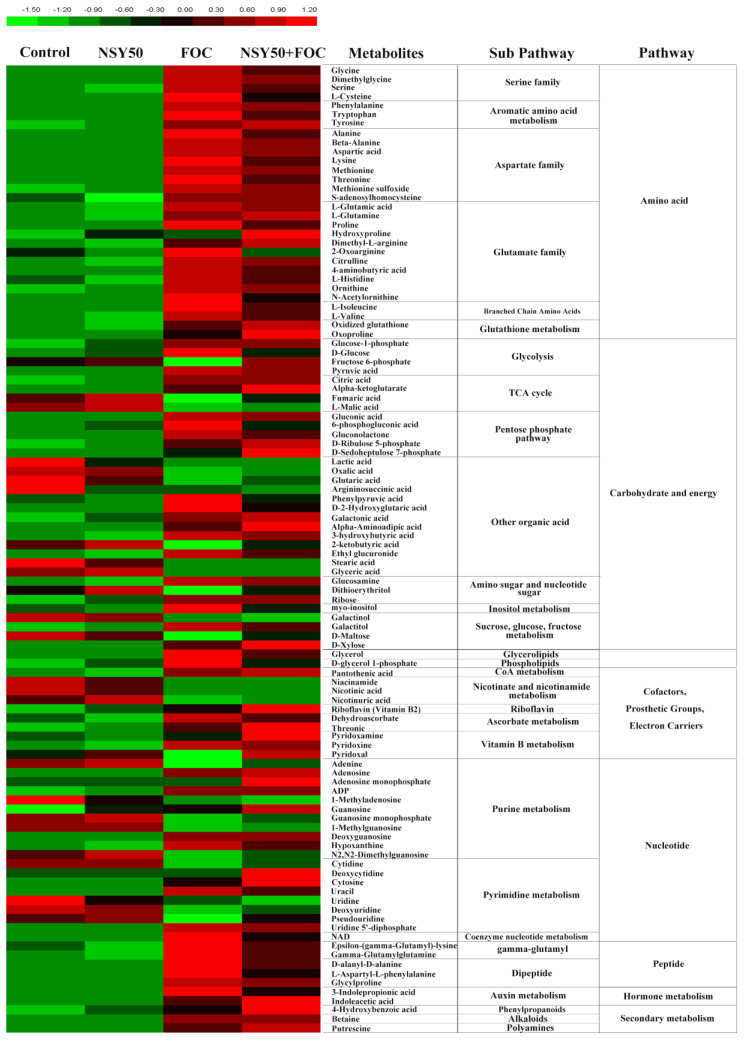
Heatmap of all of the metabolites based on relative peak area in the control and NSY50 and/or FOC-applied cucumber roots. Green (low) to red (high) represents the increase in relative area of each peak among the four treatments. This figure corresponds to Supplemental Table S2.

For untargeted metabolomic analysis, we selected a total of 140 structural metabolite identities (46 detected with GC–MS and 94 detected with LC–MS), and 106 of those metabolites (36 identified with GC–MS and 70 with LC–MS) exhibited significant variations at a *p <* 0.05 level between different treatment samples (Figure 4, Supplemental Tables S2 and S3). Compared to the control, NSY50 inoculation alone had a lesser effect on root metabolites, with eight metabolites showing increased levels and 11 presenting decreased levels (Supplemental Table S2). Furthermore, in the *F. oxysporum* treatment (FOC), the contents of 74 metabolites increased and the contents of 26 metabolites decreased compared to the control. The contents of 73 metabolites and 20 increased and decreased, respectively, in the NSY50 + FOC treatment compared to the control. Eight functional groups were clustered from the significantly different metabolites: amino acids, carbohydrates and energy, lipids, cofactors, nucleotides, peptides, hormones, and secondary metabolites (Figure 4 and Supplemental Table S2).

A total of 30 metabolites of amino acids and their derivatives were detected (17 with LC–MS and 13 with GC–MS). Except for hydroxyproline, the levels of almost all other 29 amino acids and their derivatives (such as glycine, L-cysteine, phenylalanine, methionine, proline, 4-aminobutyric acid, and GSSG) were significantly up-regulated by FOC, with values 1.57–85.18 times higher than those of the control treatment. Compared to inoculation with FOC alone, the inoculation of FOC after pretreatment with NSY50 further increased the relative contents of L-glutamine, hydroxyproline, dimethyl-L-arginine, GSSG, and oxoproline, among which hydroxyproline (NSY50 + FOC/FOC = 1.87, *p*-value = 0.08) and oxoproline (NSY50 + FOC/FOC = 1.69, *p*-value < 0.001) reached significant levels. On the contrary, under the conditions of the NSY50 + FOC treatment, glycine, serine, L-cysteine, phenylalanine, tryptophan, alanine, aspartic acid, lysine, threonine, proline, 2-oxoarginine, 4-aminobutyric acid, L-histidine, N-acetylornithine, L-isoleucine, and L-valine contents markedly decreased at *p <* 0.05 (Supplemental Table S2).

In addition to significant amino acid changes, differences in the response of the two strains to carbohydrate metabolism were observed. In particular, the pentose phosphate pathway (PPP), glycolysis, the tricarboxylic acid (TCA) cycle, and organic acid metabolites were significantly changed (Figure 4 and Supplemental Table S2). Specifically, compared to the control, the inoculation of NSY50 alone increased the contents of D-ribulose 5-phosphate, galactonic acid, and dithioerythritol, whereas the inoculation of FOC caused significant changes in the contents of 34 substances related to glycolysis, the TCA cycle, and organic acids, of which the contents of 20 substances markedly increased compared to those of the control. These 20 substances included glucose-1-phosphate, D-glucose, and pyruvic acid which are involved in glycolysis; citric acid, and alpha-ketoglutarate, which are involved in the TCA cycle; gluconic acid, 6-phosphogluconic acid gluconolactone, and D-ribulose 5-phosphate, which are involved in PPP; and several organic acids, such as phenylpyruvic acid, D-2-hydroxyglutaric acid, galactonic acid, alpha-aminoadipic acid, ethyl glucuronide, glucosamine, and galactitol (*p* < 0.05; Figure 4, Supplemental Tables S2 and S3). Furthermore, FOC significantly decreased the contents of 13 substances, such as fructose 6-phosphate, fumaric acid, L-malic acid, lactic acid, 2-ketobutyric acid, and D-maltose, indicating that FOC significantly inhibited the TCA cycle pathway, whereas PPP was enhanced compared to the control. Compared to inoculation with FOC alone, the NSY50 + FOC treatment significantly increased the contents of fructose 6-phosphate, D-ribulose, alpha-ketoglutarate, alpha-aminoadipic acid, 5-phosphate D-sedoheptulose-7-phosphate, dithioerythritol, and D-maltose and significantly decreased the contents of D-glucose, pyruvic acid, 6-phosphogluconic acid, and gluconolactone, indicating that pretreatment with NSY50 and inoculation with FOC further enhanced glycolysis, the TCA cycle, and the PPP and accelerated carbohydrate metabolism compared to inoculation with FOC alone.

In terms of other differential metabolites, such as lipids, cofactors, nucleotides, peptides, hormone metabolites, and secondary metabolites, the contents of 42 significantly changed in response to the inoculation of the two strains. Briefly, in contrast to the control, FOC inoculation markedly elevated the contents of lipids (such as glycerol and D-glycerol 1-phosphate), ascorbate metabolites (dehydroascorbic acid and threonic acid), peptides (such as epsilon-(gamma-glutamyl)-lysine, gamma-glutamyl, glutamine, D-alanyl-D-alanine, L-aspartyl-L-phenylalanine, and glycylproline), auxin metabolites (such as 3-indolepropionic acid indoleacetic acid (IAA), and secondary metabolites (such as 4-hydroxybenzoic acid, betaine, and putrescine) and decreased the contents of nicotinate and nicotinamide metabolites (such as niacinamide, nicotinic acid, and nicotinuric acid) and most of the contents of nucleotide metabolites (such as adenine, 1-methyladenosine, guanosine monophosphate, 1-methylguanosine, cytidine, uridine, deoxyuridine, and pseudouridine). FOC stress observably increased the contents of ADP and NAD to values 36.13 and 6.28 times (*p <* 0.05, Supplemental Tables S2 and S3) higher than those of the control, respectively. Furthermore, relative to the FOC treatment, NSY50 + FOC markedly increased the contents of most of the substances in nucleotide metabolism, such as riboflavin, threonic acid, pyridoxamine, adenosine monophosphate, guanosine, deoxycytidine, and cytosine and significantly decreased the contents of peptide metabolites. In contrast, NSY50 + FOC markedly increased the content of IAA, 4-hydroxybenzoic acid, and putrescine in cucumber seedling roots, in which the contents of IAA, 4-hydroxybenzoic acid, and putrescine increased by 39.3%, 52.7%, and 30.6%, respectively, compared to those of the FOC treatment (*p <* 0.05, Supplemental Table S2).

### 3.4. GSH Content and Activities of GSH-Related Enzymes in Response to NSY50 and FOC Inoculation

**Figure 5:**
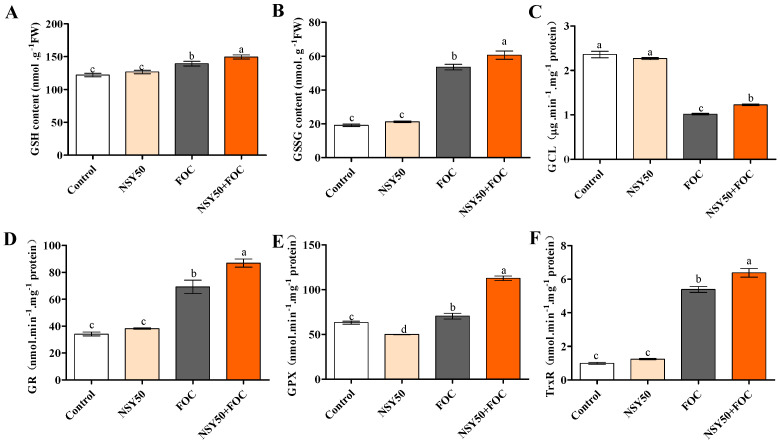
Effects of *P. polymyxa* and/or *F. oxysporum* inoculation on glutathione (GSH) redox status in the roots of cucumber seedlings. (**A**) GSH content, (**B**) GSSG content, (**C**) Activity of GCL, (**D**) Activity of GR, (**E**) Activity of GPX, (**F**) Activity of TrxR. Values are the means ± SD, *n* = 3 (biological replicates), and different letters (a > b > c > d) indicate a significant difference at *p* < 0.05, as determined by Duncan’s multiple-range test.

As depicted in Figure 5, the contents of reduced GSH and GSSG in cucumber roots significantly increased under FOC stress, with values reaching 14.21% and 180.19%, respectively, in comparison to the control. In contrast, the inoculation of FOC after NSY50 + FOC further increased GSH and GSSG contents, with values 7.23% and 13.27% higher than those inoculated with FOC alone, respectively. The primary enzymes implicated in the GSH redox cycle, such as GCL, GPX, GR, and TrxR, responded to FOC stress. Under FOC stress, the activities of GR, GPX, and TrxR were considerably greater than those of the control, increasing by 102.75%, 11.48% and 444.57%, respectively. The activity of GCL was noticeably reduced compared to that of the control group, decreasing by 56.78%. With the inoculation of FOC after NSY50 + FOC, the activities of GCL, GR, GPX and TrxR increased by 20.90%, 25.49%, 59.94%, and 18.38%, respectively, compared to those under FOC inoculation alone.

### 3.5. Expression Levels of Genes Related to GSH Metabolism

**Figure 6:**
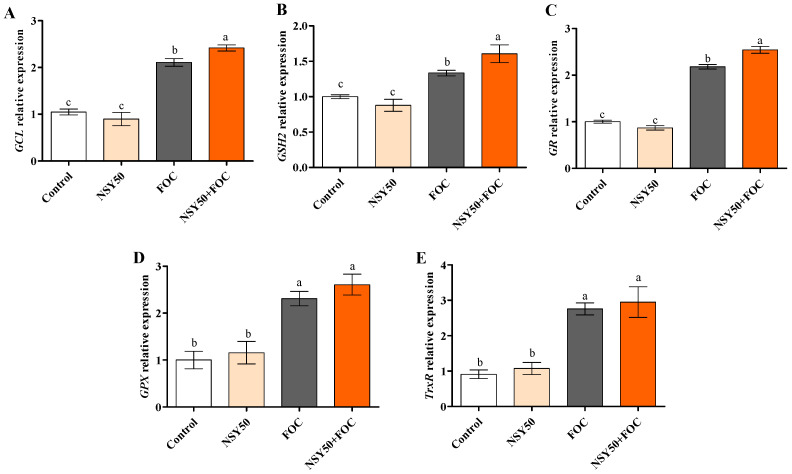
Expression of GSH biosynthesis and metabolism related genes in cucumber roots. (**A**) expression of γ-glutamylcysteine ligase, *GCL*; (**B**) expression of glutathione synthetase, *GSH2*; (**C**) expression of glutathione reductase, *GR*; (**D**) expression of Glutathione peroxidas, *GPX*; (**E**) expression of Thioredoxin reductase, *TrxR*. Root samples were harvested at three days post-inoculation (dpi) with FOC in different treatments. Each value is means ± SE of three independent experiments. Values are the means ± SD, *n* = 3 (biological replicates), and different letters (a > b > c) indicate a significant difference at *p* < 0.05, as determined by Duncan’s multiple-range test.

The further analysis of the expression of GSH cycle-related genes (see Figure 6) showed that compared to the control, *GCL* and *GSH2* genes involved in GSH synthesis were markedly up-regulated after the inoculation with FOC, increasing by 101.26% and 33.53%, respectively. Furthermore, the addition of FOC after NSY50 pretreatment further induced their expressions, increasing them by 14.65% (*GCL*) and 20.37% (*GSH2*) compared to the FOC treatment. The gene expressions of *GR*, *GPX*, and *TrxR* were markedly up-regulated, increasing by 153.91%, 130.91%, and 202.96%, respectively, in FOC inoculation seedlings compared to those of the control. During the inoculation of FOC after pretreatment with NSY50, compared to the inoculation with FOC alone, the gene expressions of *GPX* and *TrxR* increased in varying degrees, though the difference was not significant. However, the gene expression of *GR* significantly decreased by 14.29%.

## 4. Discussion

Numerous studies have reported the capability of PGPB to enhance plant growth under various conditions and to alleviate biotic stresses including Fusarium wilt [3,8,42,43,44]. Plants inoculated with these bacteria have been shown to develop long roots, increased biomass production, and enhanced resistance to the adverse effects of diverse environmental events [45]. The inhibition of growth is the most common and significant effect when plants suffer biotic and abiotic stressors [46]. In this study, the fresh and dry weights of cucumber seedlings under FOC stress were found to be significantly decreased; however, pretreatment with *P. polymyxa* NSY50 was able to effectively alleviate the growth inhibition caused by FOC stress (Figure 1).

ROS are highly reactive and toxic substances for plants. Various antioxidative defense mechanisms have been found to effectively nullify excess ROS production under steady-state conditions [47]. The balance between ROS production and scavenging can be disrupted by a variety of biotic and abiotic stressors [48,49,50]. Then, excess ROS production causes oxidative injury in plant tissues, namely the cell membrane along with proteins and DNA and RNA molecules [51]. Infection by pathogens leads to the over-production of ROS, the destruction of cell structures, and oxidative damage [52,53]. In this study, DAB and NBT staining revealed that excessive ROS accumulated in cucumber seedling roots under FOC stress (Figure 3A,B), resulting in oxidative stress and membrane lipid damage (Figure 2); these results are consistent with those of previous studies. Recently, researchers have focused their efforts on controlling Fusarium wilt through the use of plant rhizosphere growth-promoting bacteria [46]. PGPR can improve the tolerance to pathogen infections by improving the antioxidant mechanism and reducing the accumulation of ROS [3,54]. Chen et al. [55] reported that *Trichoderma harzianum* could balance the excessive production of ROS by enhancing the antioxidant capacity, reducing the H_2_O_2_ and MDA contents, and reducing the rate of O_2_^.-^ production in the roots of cucumber seedlings infected by *F. oxysporum*. Yuan et al. [56] revealed that *Pseudomonas aeruginosa* could reduce ROS accumulation and increase antioxidant enzyme activity in seedling leaves under NaCl stress. *Trichoderma* inoculation was shown to improve cucumber seedlings’ resistance to Fusarium wilt by promoting growth, boosting the antioxidant defense system, and reducing the permeability of plasma membrane and the MDA content [57]. In this study, the application of NSY50 (NSY50 + FOC) was able to markedly reduce the accumulation of MDA and O_2_^.-^ in roots under FOC stress (Figure 2), suggesting that *P. polymyxa* NSY50 could alleviate FOC-induced oxidative stress and growth inhibition in plants (Figure 1).

Biotic or abiotic stress can affect plant metabolic composition [58,59], and the ability of plant rhizosphere growth-promoting bacteria to alleviate stress is closely related to plant metabolism [60,61]. He et al. [62] indicated that phosphorus-solubilizing bacteria improved the tolerance to cadmium by regulating amino acid, organic acid, and carbon metabolism in *Solanum nigrum* roots. According to Cai et al. [63], plant rhizosphere growth-promoting bacteria can resist tomato root rot by regulating metabolic components. Our results showed that the alleviation of cucumber Fusarium wilt by the plant rhizosphere growth-promoting strain NSY50 is related to the regulation of central carbon metabolism, amino acids, and other derivative metabolites (Figure 4).

The three important components of central carbon metabolism are glycolysis, the TCA cycle, and the PPP, which are the main sources of energy for organisms and provide precursors for other metabolisms in the body [64]. Under oxidative stress conditions, plants unable to prevent the formation of ROS by adjusting central metabolic fluxes continue to maintain the biosynthesis of essential metabolites, and initiate the generation of protective compounds [65]. During stress conditions, disaccharides are hydrolyzed to glucose, which is then used in glycolysis and the TCA cycle to improve plant metabolism and resistance to stress [66,67]. In this study, the contents of xylose, inositol, and galactosyl were substantially higher in plants with Fusarium wilt stress than those of the control, and this result may have been related to plant-induced disease resistance. However, the decrease in maltose content may have been due to the hydrolysis of more maltose into glucose, which entered glycolysis, the TCA cycle, and the PPP. Under a stress environment, the carbon skeleton of glycolysis may enter into the PPP to generate NADPH for antioxidation [35,68]. The PPP-mediated recycling of NADPH is an essential antioxidant process for preventing salt-induced oxidative damage [69,70]. This finding is strongly supported by our present study, in which we observed that the content of fructose 6-phosphate (a key metabolite in glycolysis pathway) was significantly lower in plants with Fusarium wilt stress plant than that of the control. However, the contents of 6-phosphate gluconic acid, 5-phosphate ribose, and 7-phosphate heptanose in PPP significantly increased, indicating that the glycolysis pathway transferred to the PPP under Fusarium wilt stress. The pre-inoculation of NSY50 (NSY50 + FOC) further increased the accumulation of intermediate metabolites in the glycolysis pathway and the PPP, providing more energy for cucumber seedlings to resist Fusarium wilt. Most TCA cycle intermediates, as well as the organic acids, have been shown to be are increased in plants exposed to different abiotic stresses including low-temperature stress [71] and drought [67]. In our experiment, plants inoculated with *F. oxysporum* showed markedly increased citric acid and α-ketoglutaric acid contents, whereas the contents of fumaric acid and malic acid significantly decreased. However, the TCA cycle has duplicity (or amphibolic catabolism and anabolism features), and its intermediates are closely associated with a wide range of biosynthetic pathways [35]; citric acid may be more involved in the synthesis of other biological pathways, thus reducing the synthesis of fumaric acid and malic acid. The result suggested that a potential mechanism of pretreatment with NSY50 (NSY50 + FOC) on resistance improving was TCA cycle acceleration, which could provide more energy for self-defense.

Amino acids are essential for protein synthesis and act as precursors for numerous metabolites with diverse functions [72]. They significantly contribute to an increase plant stress tolerance through the promotion of the higher level accumulation of compatible osmolytes [73]. The inoculation of PGPR will lead to an increased amino acid metabolism, thus hastening plant growth [74,75]. Cai et al. [63] observed that increasing amino acid metabolism played a key role in regulating the tomato plants growth caused by plant rhizosphere growth-promoting bacteria and their resistance to pathogens. Our study revealed that numerous kinds of amino acids, especially glutamic acid, ornithine, cysteine, and glycine, significantly increased under *F. oxysporum* stress than those of control groups. However, the contents of glutamate and ornithine significantly decreased after inoculation with growth-promoting strain NSY50. This outcome may be attributed to the functional roles of glutamate metabolism in attenuating nitrogen flux, as it continues to increase carbon availability and to regulate cytosolic pH to assist plant growth [76]. Under environmental stress, glutamic acid and ornithine can be further decomposed into proline, putrescine, and other metabolites to improve resistance to the effects of adverse environment [35,77,78]. Proline is involved in the response to multiple environmental stresses [79,80]. Proline content increases after a plant becomes susceptible to diseases, and it is used as an osmotic regulator to alleviate stress [81]. This finding is also supported by our present observations, as the proline content in our study was markedly higher in *F. oxysporum* stress plants than that of the control. However, after pre-inoculation with NSY50 (NSY50 + FOC), the proline content significantly decreased, a finding that may be related to the conversion of proline to hydroxyproline. Hydroxyproline is ubiquitous in plants and plays a critical role in plant defense [82,83]. In addition, cysteine metabolism is an important pathway related to GSH synthesis, and GSH metabolism is significantly involved in plant antioxidation [84,85]. GSH accumulation is related to plant susceptibility [86], a condition supported by our results suggesting that the content of cysteine markedly increased under the infection of *F. oxysporum* but significantly decreased after inoculation with NSY50 (NSY50 + FOC). These results may be related to the increased inflow of cysteine into the GSH cycle.

The AsA-GSH cycle is a crucial element of the ROS homeostasis mechanism in plants [87], and it also plays contributes vital role in adjusting plant-pathogen interactions. Recently, special attention has been paid to explore the regulatory functions of the AsA–GSH cycle in plant defense against biotic stress [88]. As an antioxidant, GSH is essential for plant signal transduction during biotic stress [89]. Previous studies revealed that muskmelon and tomato plants’ resistance to pathogen infection was strictly related to increases in GSH and GSSH activity [90,91]. In addition, GSH is likely to exert a critical function in the regulation of various gene expressions [86,92]. However, during pathogen infection, the action mechanisms of GSSH and key enzymes (GCL, GPX, and GR) involved in the GSH redox cycle in plant defense have not been fully explored. GSH biosynthesis is controlled by the transcriptional and post-translational regulatory frameworks of GCL. Dubreuil-Maurizi et al. [93] showed that the GSH deficiency of *Arabidopsis pad2-1* mutants is related to decreases in the GCL protein level. In addition, PGPR can induce plants to resist Fusarium wilt and increase the GSH content [55,90]. Ren et al. [94] reported that PGPR could increase the contents of antioxidants, including AsA and GSH, under stress. Chen et al. [55] stated that the enhancement of cucumber root resistance to *F. oxysporum* by *Trichoderma* was due to increases in the contents of AsA and GSH in cucumber roots and the activity and gene expression of key enzymes, such as GR and MDHAR. In this study, *P. polymyxa* NSY50, a plant-rhizosphere growth promoter, induced resistance to FOC by increasing the GSH and GSSG contents in cucumber seedling roots and the activities and expressions of key enzymes (GR, GPX and GCL) involved in the GSH redox cycle. Thus, the increases in GSH and GSSG contents induced by NSY50 under FOC infection are related to the acceleration of the GSH redox cycle rate.

## 5. Conclusions

**Figure 7:**
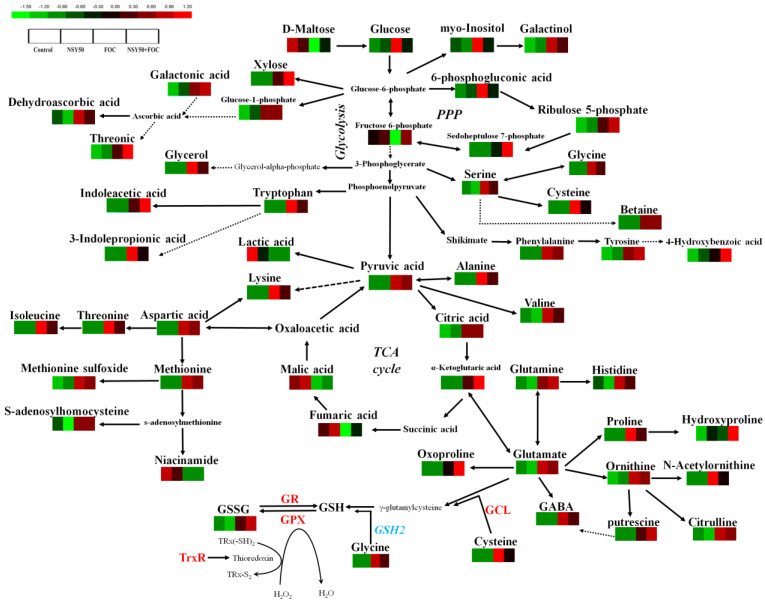
Visualization of the metabolite changes and related pathways in *P. polymyxa* NSY50 treated cucumber plants (NSY50), NSY50-pretreated cucumber plants challenged with FOC strain (NSY50 + FOC), FOC treated cucumber plants (FOC), and sterile distilled water-treated control (Control). All of the metabolites based on relative peak area in the control and strains NSY50 and FOC-applied cucumber roots. Green (low) to red (high) represents the increase in relative area of each peak among the four treatments. This figure corresponds to Table S2. Significant difference at *p <* 0.05. Abbreviations: GCL, γ-glutamylcysteine synthase; GR, Glutathione reductase; GPX, glutathione peroxidase; TrxR, thioredoxin reductase.

In summary, our results demonstrated that the supplementation of *P. polymyxa* NSY50 to cucumber alleviated the inhibitory effect of *F. oxysporum* on growth and enhanced the tolerance of plants to Fusarium wilt. Metabolomic analysis revealed that root glycolysis and the PPP transferred to the TCA cycle under Fusarium wilt stress, whereas NSY50 + FOC reduced the adverse effects of glycolysis and the TCA cycle and promoted energy metabolism. In addition, NSY50 pretreatment activated the GSH cycle and increased the antioxidant capacity (Figure 7). This study enriches our understanding of PGPR-mediated plant stress response mechanism and promotes the protection of plants against pathogens.

## Data Availability

Data are contained within the article and Supplementary Material.

## Supplementary Materials

The following supporting information can be downloaded at: https://www.mdpi.com/article/10.3390/biology11071028/s1. Supplementary Figure S1. Effect of GSH biosynthesis inhibitor buthionine sulfoximine (BSO) on Fusarium wilt responses in cucumber seedlings pretreated with NSY50. (A) and (B) Phenotype of cucumber seedlings. (C) Disease index for 2 works post-inoculation. (D) Fresh weight. (E) and (F) H_2_O_2_ and MDA contents for 3 days post-inoculation. (G) GSH contents for 3 days post-inoculation. (H) GR activity for 3 days post-inoculation. Values are the means ± SD, *n* = 3 (biological replicates). Reference [95] is mentioned in Supplementary Materials. Supplementary Table S1. Sequences of gene-specific primers in quantitative real-time polymerase chain reaction (qRT-PCR) analysis. Supplementary Table S2. Heatmap and tabular presentation of all metabolites based on relative peak area in the control and NSY50 and/or FOC-applied cucumber roots. Supplemental Table S3. Functional classification and quantitative analysis of major metabolites in the control and NSY50 and/or FOC-applied cucumber roots using GC-Ms and HPLC-MS. Metablites exhibiting exhibited significant quantitative changes were highlighted in yellow.
