# Supplementary material for: Dissection of Paenibacillus polymyxa NSY50-Induced Defense in Cucumber Roots against Fusarium oxysporum f. sp. cucumerinum by Target Metabolite Profiling
Source: Biology (Basel). 2022 Jul 8;11(7):1028. doi: 10.3390/biology11071028 (PMC9311960; doi:10.3390/biology11071028)
Supplement: Supplementary file 1 [file biology-11-01028-s001.zip › Supplementary Figure S1.pdf]

Supplementary Figure S1

Dissection of *Paenibacillus polymyxa* NSY50 -induced defense in cucumber roots against *Fusarium oxysporum* f. sp. *cucumerinum* by target metabolite profiling

1、This set of experiment was conducted to confirm the role of glutathione (GSH) in the *Paenibacillus polymyxa* NSY50-triggered tolerance against cucumber Fusarium wilt.

Paenibacillus polymyxa-NSY50 was grown on LB medium at 28℃ for 3 days, and cell suspensions were adjusted to a cell density of 10^8 cells per mL. The cucumber *Fusarium* wilt pathogen *Fusarium oxysporum* f. sp. *cucumerinum* (FOC) was incubated in PDB liquid culture for 7 days, then filtered through two layers of cheesecloth and adjusted to 1×10^7 conidia/mL with sterile distilled water as determined by a hemacytometer count.

Healthy cucumber seeds (*Cucumis sativus* L. cv. "Jincheng NO.4") were grown in 50-hole seedling tray filled with vermiculite. When the cotyledons were fully expanded, the seedlings were placed in 1-L plastic containers (six seedlings per containers) containing half-strength Hoagland nutrient solution (pH 6.5±0.1, EC 2.0–2.2 mS·cm^{-1}).The seedlings were grown in a light growth chamber in Henan Agricultural University, that was maintained at 28℃ (day) and 25°C (night) with 65-75% relative humidity (RH), a light intensity of 200 μmol m^{-2}s^{-1}, and a photoperiod of 14-h day/10-h night. One-leaf stage cucumber seedlings were applied to the following experiments.

We performed assays included five treatments:(1) Control, control plants were grown in Hoaland's solution; (2) NSY50, seedlings grown in Hoagland nutrient solution containing 100 mL 1.0×10^8 NSY50 cell suspension; (3) FOC: 100 mL of a cell suspension of FOC at 1×10^7 conidia/ml was poured into the tanks after 3d of per-culture; (4) NSY50+FOC: inoculated with 100ml of NSY50 (1×10^8 CFU/mL) of per-culture, then 3d later, challenge-inoculated with 100 ml of a cell suspension of FOC (1×10^7 conidia/mL)；(5) NSY50+FOC+BSO: cucumber seedlings were firstly grown in Hoagland solution containing with 100 μM BSO for 48 h，then inoculated with 100ml of NSY50 (1×10^8 CFU/mL) after 3d, and were finally challenge-inoculated with 100 ml of a cell suspension of FOC (1×10^7 conidia/mL).BSO is a well-known inhibitor of GSH biosynthesis [95].

3 days after inoculation of the pathogen, the content of malondialdehyde (MDA) in roots of cucumber was determined as thiobarbituric acid reactive substances (TBARSs) formation according to the method of Liu et al. [29]. H_2O_2 contents in roots of cucumber was determined as described by Yuan et al. [31]. The content of glutathione was determined according to Zhong et al. [37]. GR activity was assayed according to Halliwell and Foyer [38] . 2 weeks after inoculation of the pathogen,the fresh weight of plant was measured with three biological replicates after washed and dried with gauze.and the disease severity was monitored based on the disease severity index scale of 0-3 (0 = healthy; 1 ≤50% of leaves wilted; 2 ≥50% of leaves wilted but plants not dead; 3 = dead plants). The disease severity index was calculated using the following formula: Disease severity

index (%) = [∑(Disease rating Number of seedlings with disease rating)/Total number of seedling× highest rating] ×100%.

A

B

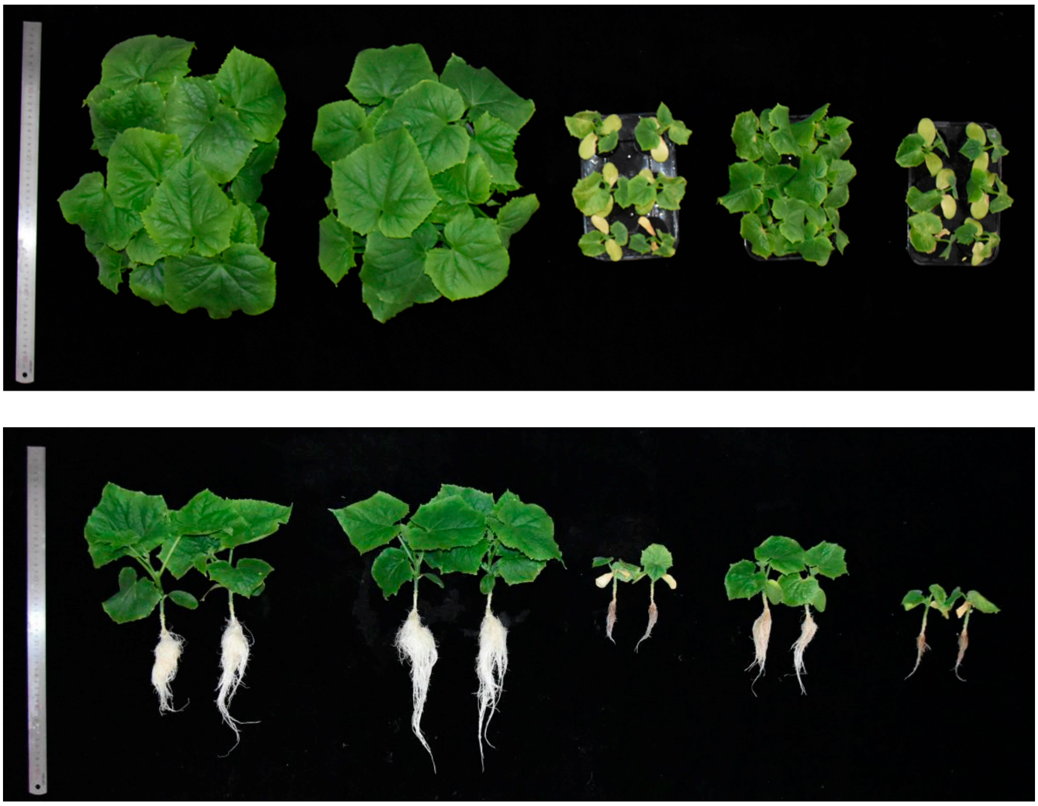

Control NSY50 FOC NSY50+FOC BSO+NSY50+FOC

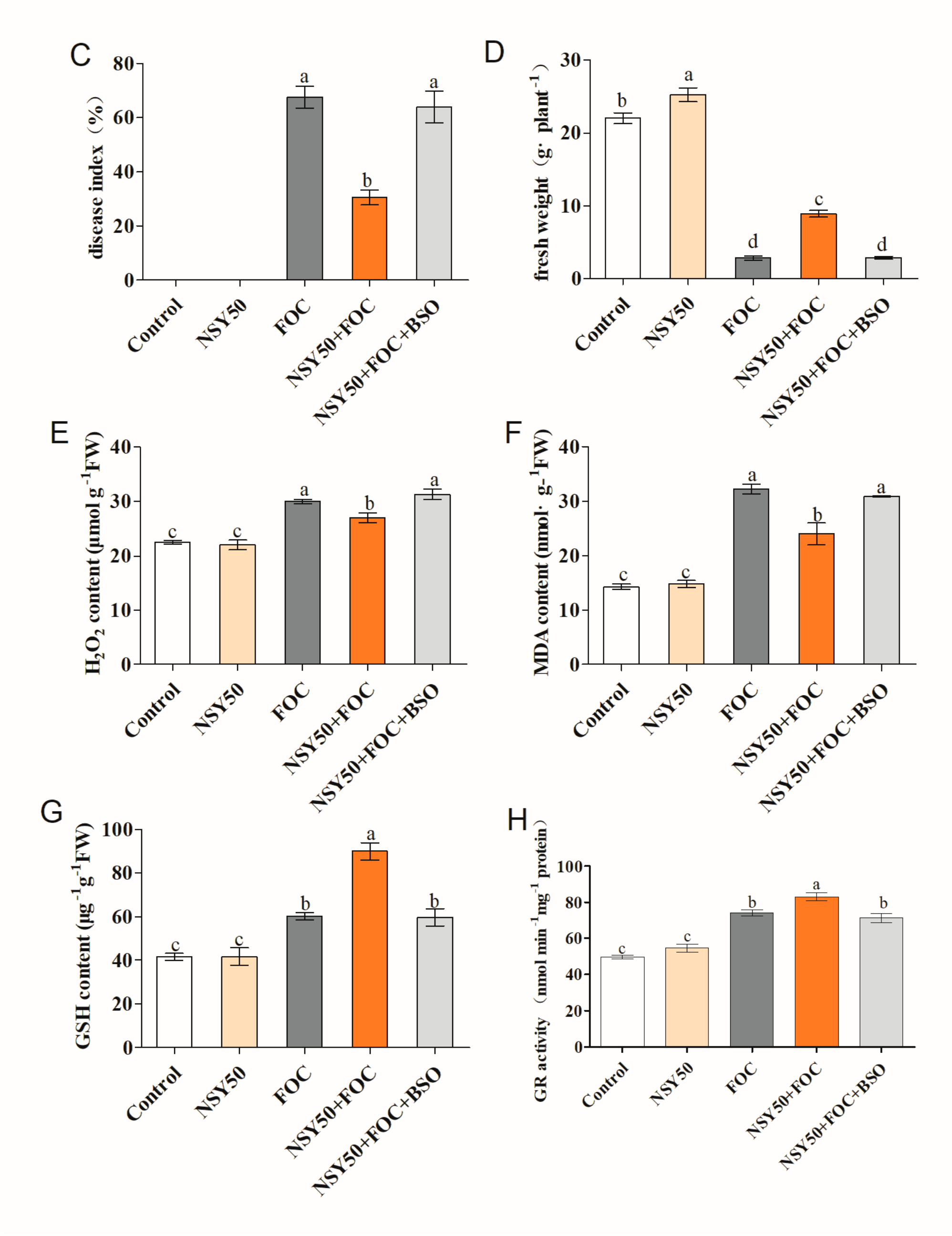

Supplementary Figure S1. Effect of GSH biosynthesis inhibitor buthionine sulfoximine (BSO) on Fusarium wilt responses in cucumber seedlings pretreated with NSY50. (A) and (B) Phenotype of cucumber seedlings. (C) disease index for 2works post-inoculation. (D) Fresh weight. (E) and (F) H_2O_2 and MDA contents for 3 days post-inoculation. (G) GSH contents for 3 days post-inoculation. (H) GR activity for 3 days post-inoculation. Each histogram represents the mean±SE of three independent biological experiments (n=3). Different letters above the bars indicate statistically significant differences by Duncan's test (*P<0.05*).

References

95. Berglund, T.; Ohlsson, A.B. The Glutathione Biosynthesis Inhibitor Buthioninesulfoximine (BSO) Induces Cardenolide Accumulation in *Digitalis lanata* Tissue Culture. *J. Plant Physiol*. 1993, 142, 248–250.
