# Supplementary material for: Dissection of Paenibacillus polymyxa NSY50-Induced Defense in Cucumber Roots against Fusarium oxysporum f. sp. cucumerinum by Target Metabolite Profiling
Source: Biology (Basel). 2022 Jul 8;11(7):1028. doi: 10.3390/biology11071028 (PMC9311960; doi:10.3390/biology11071028)
Supplement: Supplementary file 1 [file biology-11-01028-s001.zip › Supplementary Table S1.pdf]

Supplementary Table S1

Sequences of gene-specific primers in quantitative real-time polymerase chain reaction (qRT-PCR) analysis

Gene family	Gene Accession No.	Annealing temperature/ºC	Forward primer (5`- 3`)	Reverse primer (5`-3`)	Length of product
actin	XM_011659465.2	58.0	CAGGAATCCACGAAACTACT	AGACCCTCCAATCCAAACAC	218
GCL	NC_026659.1	58.0	AGGTAGGGTGATTGTTGCTG	TCCATTTTTCCTTAGGCTTG	122
GSH2	NC_026655.1	58.0	ACAATGGCGGATGTTCATGG	TCCTGGTACTTCGCCTGATC	132
GR	NM_001308836.1	58.0	CTAAGCGTGTTGTGGTGCTT	ACTTTGGCACCCATACCATT	79
GPX	FJ036896.1	58.0	ATACAAGGGCCACGGATTTG	CTTGATGGCGTCTCCAAAGAG	217
TrxR	XM_031883233.1	58.0	AGGGAATGAGAATAATAATAACG	GCGGAAGATATTGGAGAA	120

Note: *actin*, housekeeping gene; *GCL*, γ -glutamylcysteine synthetase (catalyses the first step of GSH synthesis); *GSH2*, glutathione synthetase (responsible for the second step of GSH synthesis); *GR*, glutathione reductase; *GPX*, Glutathione peroxidase; *TrxR*, Thioredoxin reductase.
