# Supplementary material for: Dissection of Paenibacillus polymyxa NSY50-Induced Defense in Cucumber Roots against Fusarium oxysporum f. sp. cucumerinum by Target Metabolite Profiling
Source: Biology (Basel). 2022 Jul 8;11(7):1028. doi: 10.3390/biology11071028 (PMC9311960; doi:10.3390/biology11071028)
Supplement: Supplementary file 1 [file biology-11-01028-s001.zip › Supplementary Table S2.pdf]

Supplementary Table S2 Heatmap and tabular presentation of all metabolites based on relative peak area in the control and NSY50 and/or FOC-applied cucumber roots.

Metabolites	Mean Absolute Peak Values				Platform	Standardized Relative Area			
	Control	NSY50	FOC	NSY50+FOC		Control	NSY50	FOC	NSY50+FOC
glycine	66.630	55.360	366.750	268.220	GC/MS	-0.799	-0.872	1.156	0.514
Dimethylglycine	23.830	20.820	114.700	99.137	LC/MS	-0.828	-0.889	1.016	0.701
serine	7.990	6.220	33.340	25.762	GC/MS	-0.775	-0.907	1.125	0.557
L-Cysteine	4.779	6.676	407.096	196.178	LC/MS	-0.778	-0.768	1.325	0.222
phenylalanine	21.140	20.040	70.410	59.701	GC/MS	-0.832	-0.875	1.059	0.648
Tryptophan	1.470	1.070	7.640	4.707	GC/MS	-0.732	-0.861	1.273	0.320
tyrosine	33.700	34.850	55.610	56.896	GC/MS	-0.910	-0.819	0.814	0.915
alanine	128.390	125.260	718.210	438.417	GC/MS	-0.788	-0.799	1.285	0.302
Beta-Alanine	13.725	13.780	25.401	22.941	LC/MS	-0.859	-0.850	1.056	0.653
aspartic acid	9.330	7.730	41.860	34.989	GC/MS	-0.809	-0.900	1.051	0.658
lysine	53.140	51.760	162.170	121.480	GC/MS	-0.812	-0.837	1.200	0.449
methionine	18.100	18.300	48.180	42.988	GC/MS	-0.865	-0.852	1.021	0.696
threonine	12.090	11.150	70.670	45.984	GC/MS	-0.795	-0.827	1.240	0.382
Methionine sulfoxide	15.040	17.730	37.420	33.414	LC/MS	-0.973	-0.732	1.032	0.673
S-adenosylhomocysteine	422.230	261.970	663.790	685.439	LC/MS	-0.424	-1.214	0.766	0.872
L-Glutamic acid	143.481	123.008	538.216	482.775	LC/MS	-0.814	-0.907	0.987	0.734
L-Glutamine	7.388	5.162	26.939	28.088	LC/MS	-0.773	-0.953	0.816	0.910
proline	46.580	40.690	235.570	162.406	GC/MS	-0.790	-0.853	1.208	0.435
Hydroxyproline	219.017	352.238	296.396	552.656	LC/MS	-0.954	-0.020	-0.412	1.386

Dimethyl-L-arginine	8041.426	6302.187	12505.550	14789.949	LC/MS	-0.604	-1.048	0.535	1.118
2-Oxoarginine	44.357	37.441	62.817	39.979	LC/MS	-0.156	-0.759	1.453	-0.538
citrulline	86.039	48.251	233.387	216.796	LC/MS	-0.648	-1.055	0.941	0.762
4-aminobutyric acid	372.480	400.800	1624.100	1179.368	GC/MS	-0.850	-0.804	1.189	0.465
L-Histidine	97.003	71.013	175.959	149.262	LC/MS	-0.550	-1.093	1.100	0.542
Ornithine	101.356	105.205	178.593	169.509	LC/MS	-0.909	-0.815	0.972	0.751
N-Acetylornithine	23.821	16.272	95.621	55.107	LC/MS	-0.662	-0.871	1.327	0.205
L-Isoleucine	254.858	230.169	628.427	445.993	LC/MS	-0.726	-0.859	1.283	0.302
L-Valine	17.539	15.343	45.141	35.328	LC/MS	-0.753	-0.906	1.172	0.488
Oxidized glutathione	21063.666	15795.122	35622.764	44048.865	LC/MS	-0.620	-1.025	0.499	1.147
oxoproline	81.200	54.600	205.350	346.385	GC/MS	-0.679	-0.878	0.250	1.306
Glucose-1-phosphate	333.546	825.909	1789.956	1768.625	LC/MS	-1.173	-0.490	0.846	0.817
D-Glucose	106.832	116.752	169.280	128.333	LC/MS	-0.855	-0.494	1.421	-0.072
Fructose 6-phosphate	160.005	174.320	120.105	179.838	LC/MS	0.053	0.584	-1.426	0.789
Pyruvic acid	4.573	4.771	16.981	14.703	LC/MS	-0.872	-0.842	1.032	0.682
citric acid	17.780	22.930	42.620	41.850	GC/MS	-1.055	-0.653	0.884	0.824
alpha-ketoglutarate	0.290	0.280	3.090	5.609	GC/MS	-0.791	-0.795	0.302	1.285
Fumaric acid	7.580	8.220	4.150	6.451	GC/MS	0.547	0.905	-1.369	-0.084
L-Malic acid	30.640	32.480	6.130	7.845	GC/MS	0.799	0.928	-0.924	-0.803
gluconic acid	0.870	1.110	32.720	28.968	GC/MS	-0.870	-0.856	0.971	0.754
6-phosphogluconic acid	3.350	5.970	29.130	9.699	GC/MS	-0.743	-0.519	1.462	-0.200
Gluconolactone	265.015	264.362	704.847	586.501	LC/MS	-0.844	-0.847	1.109	0.583
D-Ribulose 5-phosphate	6.285	10.666	23.230	32.007	LC/MS	-1.001	-0.628	0.441	1.188
D-Sedoheptulose	2.552	2.571	19.093	54.000	LC/MS	-0.701	-0.700	-0.019	1.420

7-phosphate									
lactic acid	2.120	0.910	0.570	0.553	GC/MS	1.462	-0.173	-0.633	-0.656
oxalic acid	9.090	8.830	4.870	5.671	GC/MS	0.915	0.795	-1.041	-0.669
Glutaric acid	5017.082	3795.738	1819.948	2746.772	LC/MS	1.215	0.328	-1.108	-0.435
Argininosuccinic acid	172.283	103.834	111.450	100.866	LC/MS	1.487	-0.542	-0.316	-0.629
Phenylpyruvic acid	35.004	28.662	76.910	45.966	LC/MS	-0.543	-0.839	1.414	-0.031
D-2-Hydroxyglutaric acid	23.702	17.123	197.898	111.806	LC/MS	-0.750	-0.827	1.293	0.284
Galactonic acid	1837.277	2599.617	3922.202	4417.391	LC/MS	-1.144	-0.501	0.614	1.031
alpha-Aminoadipic acid	0.280	0.350	1.280	2.066	GC/MS	-0.842	-0.760	0.337	1.265
3-hydroxybutyric acid	0.210	0.170	0.890	0.824	GC/MS	-0.811	-0.915	0.948	0.778
2-ketobutyric acid	7.680	8.210	4.320	6.392	GC/MS	0.595	0.901	-1.346	-0.150
Ethyl glucuronide	996.810	678.680	4823.460	3360.958	LC/MS	-0.743	-0.904	1.194	0.453
stearic acid	1.900	1.340	0.710	0.619	GC/MS	1.266	0.331	-0.722	-0.875
Glyceric acid	14.598	16.808	6.587	6.717	LC/MS	0.645	1.062	-0.866	-0.841
Glucosamine	10.600	6.230	32.640	28.644	LC/MS	-0.684	-1.018	1.004	0.698
Dithioerythritol	28.730	38.470	12.240	26.266	GC/MS	0.213	1.112	-1.310	-0.015
ribose	7.710	8.680	10.480	10.637	GC/MS	-1.172	-0.490	0.776	0.886
myo-inositol	10.890	10.310	18.170	12.121	GC/MS	-0.549	-0.710	1.467	-0.208
galactinol	1610.350	1475.840	691.860	587.095	GC/MS	0.986	0.731	-0.759	-0.958
Galactitol	44.160	56.930	219.040	158.309	LC/MS	-0.902	-0.749	1.188	0.462
D-Maltose	14842.340	12590.620	7602.420	10442.017	LC/MS	1.125	0.396	-1.220	-0.300
D-Xylose	16.610	16.530	21.800	25.807	LC/MS	-0.797	-0.815	0.360	1.253
glycerol	123.010	120.930	395.350	291.039	GC/MS	-0.814	-0.829	1.209	0.434
D-(glycerol 1-phosphate)	4.660	6.870	12.940	7.755	GC/MS	-0.969	-0.338	1.393	-0.086

Pantothenic acid	878.420	775.490	1764.500	1918.923	LC/MS	-0.772	-0.946	0.728	0.990
Niacinamide	528.030	378.890	43.750	37.900	LC/MS	1.142	0.536	-0.827	-0.851
Nicotinic acid	64.100	56.150	40.680	41.569	LC/MS	1.177	0.483	-0.869	-0.791
Nicotinuric acid	74.090	90.910	38.180	43.387	LC/MS	0.495	1.164	-0.933	-0.726
riboflavin (Vitamin B2)	12427.660	15713.860	23302.320	33618.720	LC/MS	-0.939	-0.590	0.216	1.313
Dehydroascorbic acid	798.450	658.390	1205.948	1080.224	LC/MS	-0.546	-1.103	1.075	0.575
Threonic	12.200	15.440	24.610	33.076	GC/MS	-0.968	-0.625	0.348	1.245
Pyridoxamine	59.500	43.910	60.460	109.248	LC/MS	-0.310	-0.860	-0.276	1.445
Pyridoxine	35.870	31.350	86.820	73.375	LC/MS	-0.764	-0.929	1.091	0.602
pyridoxal	97.710	108.740	69.730	120.626	LC/MS	-0.069	0.438	-1.354	0.984
adenine	5300.246	5623.404	3566.484	4392.064	LC/MS	0.624	0.972	-1.242	-0.353
adenosine	6991.854	6653.676	17932.216	18884.993	LC/MS	-0.839	-0.890	0.793	0.935
Adenosine monophosphate	599.995	594.747	609.977	849.622	LC/MS	-0.512	-0.554	-0.432	1.498
ADP	1.248	6.488	45.097	46.477	LC/MS	-0.970	-0.755	0.834	0.891
1-Methyladenosine	18662.972	14168.550	10651.551	9483.507	LC/MS	1.314	0.225	-0.628	-0.911
guanosine	369.158	501.893	549.286	671.445	LC/MS	-1.231	-0.168	0.211	1.188
Guanosine monophosphate	3450.272	3971.480	1348.456	2165.180	LC/MS	0.599	1.035	-1.159	-0.476
1-Methylguanosine	417.833	423.571	45.582	97.951	LC/MS	0.847	0.875	-0.990	-0.732
Deoxyguanosine	375.968	374.320	2349.681	2372.236	LC/MS	-0.865	-0.867	0.856	0.876
Hypoxanthine	1759.950	1220.570	4157.170	3209.114	LC/MS	-0.616	-1.018	1.170	0.464
N2,N2-Dimethylguanosine	199.328	285.241	29.982	98.012	LC/MS	0.412	1.177	-1.097	-0.491
cytidine	57362.030	55542.430	6841.573	21017.345	LC/MS	0.879	0.807	-1.123	-0.562
Deoxycytidine	3490.360	3467.250	5613.305	19670.277	LC/MS	-0.586	-0.588	-0.314	1.487
Cytosine	414.750	338.500	664.842	1127.111	LC/MS	-0.623	-0.837	0.080	1.380

uracil	9.370	10.320	50.020	37.548	GC/MS	-0.861	-0.815	1.146	0.530
uridine	423.128	328.337	289.926	251.978	LC/MS	1.358	0.068	-0.455	-0.971
Deoxyuridine	15.380	14.670	9.278	10.920	LC/MS	0.960	0.718	-1.118	-0.559
Pseudouridine	32.360	35.840	11.610	27.579	LC/MS	0.515	0.840	-1.423	0.068
Uridine 5'-diphosphate	190.379	203.311	540.380	503.026	LC/MS	-0.897	-0.828	0.962	0.764
NAD	13.236	10.484	83.117	36.615	LC/MS	-0.673	-0.755	1.406	0.022
Epsilon-(gamma-Glutamyl)-lysine	52.260	42.312	79.017	65.833	LC/MS	-0.475	-1.096	1.197	0.374
Gamma-Glutamylglutamine	64.470	36.510	277.060	176.475	LC/MS	-0.672	-0.926	1.255	0.343
D-alanyl-D-alanine	4.470	4.380	12.750	9.566	GC/MS	-0.810	-0.832	1.210	0.433
L-Aspartyl-L-phenylalanine	5.915	6.461	211.953	103.272	LC/MS	-0.775	-0.769	1.327	0.218
Glycylproline	31.768	41.012	331.428	305.038	LC/MS	-0.892	-0.836	0.945	0.783
3-Indolepropionic acid	19.623	15.952	124.137	53.346	LC/MS	-0.671	-0.744	1.413	0.002
Indoleacetic acid	47.660	45.033	129.190	179.912	LC/MS	-0.802	-0.842	0.437	1.207
4-Hydroxybenzoic acid	1.300	1.980	2.930	4.478	GC/MS	-0.996	-0.502	0.187	1.311
Betaine	2085.530	1424.540	29195.060	28894.158	LC/MS	-0.845	-0.887	0.875	0.856
